# Tumor-Associated Glycans and Their Role in Gynecological Cancers: Accelerating Translational Research by Novel High-Throughput Approaches

**DOI:** 10.3390/metabo2040913

**Published:** 2012-11-14

**Authors:** Tatiana Pochechueva, Francis Jacob, Andre Fedier, Viola Heinzelmann-Schwarz

**Affiliations:** Gynecological Research Group, Department of Biomedicine, University Hospital Basel, Hebelstrasse 20, 4031 Basel, Switzerland

**Keywords:** carbohydrate, malignancy, anti-glycan antibodies, tumor marker, microarray, glycomics, breast cancer, ovarian cancer

## Abstract

Glycans are important partners in many biological processes, including carcinogenesis. The rapidly developing field of functional glycomics becomes one of the frontiers of biology and biomedicine. Aberrant glycosylation of proteins and lipids occurs commonly during malignant transformation and leads to the expression of specific tumor-associated glycans. The appearance of aberrant glycans on carcinoma cells is typically associated with grade, invasion, metastasis and overall poor prognosis. Cancer-associated carbohydrates are mostly located on the surface of cancer cells and are therefore potential diagnostic biomarkers. Currently, there is increasing interest in cancer-associated aberrant glycosylation, with growing numbers of characteristic cancer targets being detected every day. Breast and ovarian cancer are the most common and lethal malignancies in women, respectively, and potential glycan biomarkers hold promise for early detection and targeted therapies. However, the acceleration of research and comprehensive multi-target investigation of cancer-specific glycans could only be successfully achieved with the help of a combination of novel high-throughput glycomic approaches.

## 1. Introduction

In this review, we provide an overview of well described tumor-associated glycans in gynecological cancers, in particularly ovarian and breast cancers, as the most common and lethal cancers in women, respectively. In addition, we link tumor associated carbohydrates (TAC) to antigenicity and its recognition by the immune system via detection of naturally occurring anti-glycan antibodies. Most of these findings were based on classical studies, such as immunohistochemical staining and ELISA, but with the recent development of glycomic microarray platforms, such as printed glycan array (PGA), glycopeptide array, surface plasmon resonance (SPR) array, suspension array and others, this research has grown rapidly. However, the possible biochemical mechanisms of action of cancer-associated glycans in cancer progression are still under evaluation and are not part of this review, where we will focus more on their occurrence in gynecological cancers and their clinical relevance.

### 1.1. Glycans and Cancer

**Figure 1:**
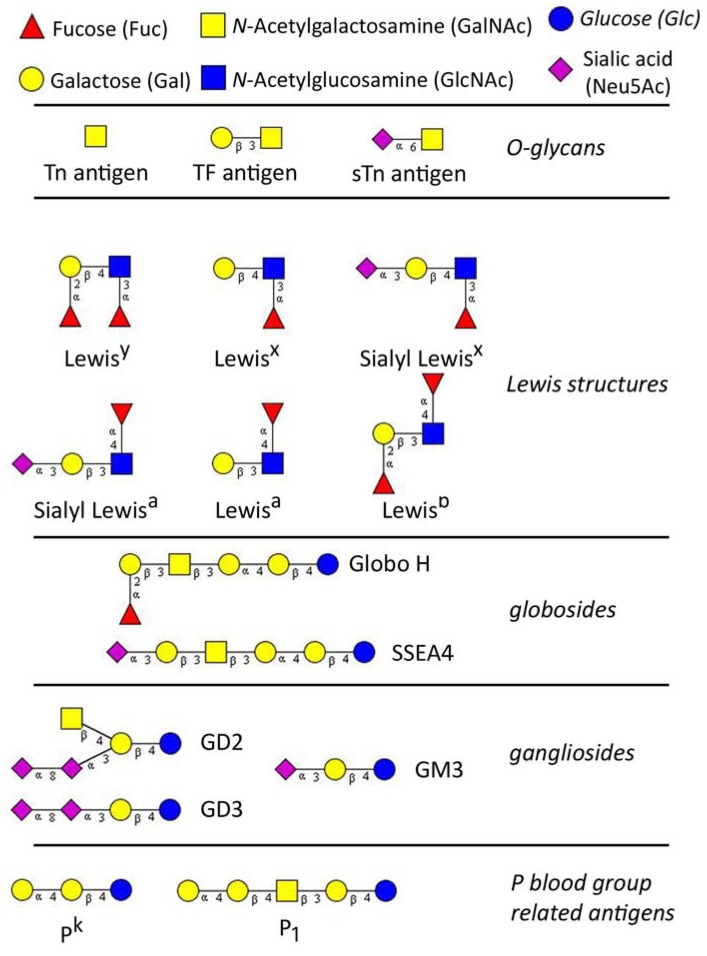
The major tumor-associated glycan determinants, reported to be involved in gynaecological cancers. Glycan structures were designed using GlycoWorkbench [18].

Glycans (carbohydrates) are poly- or oligosaccharides, homo- or heteropolymers of monosaccharide residues, and important partners in many biological processes including carcinogenesis. Aberrant glycosylation of proteins and lipids occurs commonly during malignant transformation and leads to the expression of tumor-specific glycans [1,2]. The alterations in glycosylation develop very early during carcinogenesis, before any destructive changes in proliferation/apoptosis or cell differentiation become discernible [3]. Tumor-associated carbohydrates (TAC) are expressed by both tumor and host cells and are involved in the key pathophysiological processes during the various steps of tumor progression, including tumor growth, cell migration, invasion, metastasis, angiogenesis, and evasion of innate immunity [4,5,6,7,8,9]. In past three decades TAC were studied extensively for the use as specific tumor biomarkers and potential therapeutic targets, however, their biological role and functional mechanisms remain still unknown. The classically known TAC are sialyl- Lewis^a ^(sLe^a^), T (or TF, Thomsen-Friedenreich) antigen and Thomsen-nouvelle antigen (Tn), an unsubstituted *GalNAc*. Basically, TAC can be divided into three major groups: (A) glycosphingolipids of the ganglio- and globo-series; (B) modified lacto-series type 1 (*Galβ1-3GlcNAc*) or type 2 chains (*Galβ1-4GlcNAc*), and (C) core glycan structures of *O*-linked mucin type (T and Tn antigens) [10,11] (Figure 1). 

The appearance of aberrant glycan structures, characterized by truncations, increased branching, altered sialylation and/or fucosylation on cancerous cells is typically associated with clinicopathological characteristics such as grade, metastasis and poor prognosis overall. In contrast, some TAC show opposite effects suppressing invasiveness and metastatic potential [2,12]. These aberrantly expressed glycans may reflect alterations of the cognate glycosyltransferase and/or glycosidase network and, initially, dysregulation of the enzyme expression [13,14]. Patterns of glycosyltransferase activities in cancer cell lines indicated that various cancer cells express certain glycan epitopes which could have diagnostic values or serve as treatment targets [10,15]. Particularly sialyltransferases and sulfotransferases may play a substantial role in the alteration of glycan performance in cancer cells and have been proposed as prognostic markers in breast cancer patients [16,17].

### 1.2. Naturally Occurring Anti-Glycan Antibodies

Anti-glycan antibodies have been shown to be disease-specific, for instance in Crohn’s disease [19,20], rheumatoid arthritis [21], infections [22] and cancer [23,24,25]. These potential anti-glycan antibodies hold therefore promise for disease-specific biomarkers and tumor markers for early cancer diagnostics. Moreover, antibodies against several tumor-associated carbohydrate antigens (TACA) have consistently been observed in human sera [26,27,28]. Autoantibodies against TACA presumably develop early in carcinogenesis when TACA appear in pre-malignant and malignant lesions. With the help of sensitive novel high-throughput platforms, such as glycopeptide arrays, anti-TACA antibodies can be detected in sera long before the antigen [23], and could provide a screening tool for early detection and prognostic assessment. 

Unfortunately, only a limited number of human anti-TACA antibodies have been evaluated for their significance in carcinogenesis. For example, an extensive study performed in patients with gastric, colon, rectal and breast cancer revealed that anti-TACA antibodies of IgM subclass against five known cancer antigens bind to carbohydrates on tumor-specific receptors and contribute to apoptosis, possibly playing an immuno-surveillance role [29]. In another study, naturally occurring antibodies against certain gangliosides and glycosphingolipids have been correlated with improved survival in melanoma and were suggested for carbohydrate vaccine design [30]. Today, the new era of glycomics using microarray-based platforms allow the first insight into yet unknown interactions of glycans and naturally occurring anti-glycan antibodies.

### 1.3. High-Throughput Technologies to Map Glycan-Antibody Interactions

Similar to protein research, the standard for investigations into anti-glycan antibodies is both custom-made [26,27,31,32,33,34,35] and commercial ELISA [36,37,38,39]. Glycans are usually bound to a carrier (BSA, polyacrylamide), forming glycoconjugates, which are attached non-covalently to a microplate surface. Despite cost-effectiveness the major disadvantage of conventional ELISA is low throughput. Based on former research technologies in transcriptomics and proteomics, glycan microarrays are now new and promising tools allowing the simultaneous detection of glycan-protein interactions. Based on this technology, we have gained insight into endogenous biological processes, microbe-host interactions, and immune defense mechanisms. 

Since the introduction of the first glycan-based arrays [40,41] the number of platform variations have continuously increased (summarized in Table 1). The glycan-based arrays are usually incorporating a glycan library which could be constructed from chemically/enzymatically synthesized or natural glycans. Glycan arrays are typically characterized by the fabrication where glycans are covalently attached via a reactive group such as amine [19,40,41], linked to a chemical spacer. Attempts have been made to avoid the use of a linker by covalently- and site-specifically immobilizing carbohydrates onto hydrazine-coated glass slides [42]. This type of platform maps glycan-protein interactions in a monomeric form. Such platforms vary in ligand presentation, density, glycan origin, assay conditions, and immobilization on flat surfaces. All this may influence glycan recognition processes. The possible limitations of glycan arrays might be a restricted flexibility in terms of assay reconfiguration and monomeric presentation of glycans on the array.

**Table 1:** Characteristics of major glycan-based array platforms.

Array type	Ref.	Glycan-presentation	Assay dynamics	Immobilization (theoretically)	Serum dilution	detection
ELISA (Glycominds)	[36,37,38,39,43,44,45]	n/a	Static	n/a	1:101	Anti-human IgA, IgG or IgM separately and each HRPO conjugated
ELISA	[26,27,31,33,34,35]	polymeric	Static	Non-covalent and non site-specific	1:25 (up to 1250	Anti-human Ig (IgA+IgG+IgM), IgG and IgM HRPO conjugated, IgM and IgG both AP conjugated;
ELISA	[32]	polymeric	Static	n/a	Undiluted up to 1:10000	Anti-human IgD, IgG2 and IgM and anti-mouse IgG HRPO conjugated
SPR array	[46]	Monomeric/polymeric	Flow	Covalent and site-specific	1:50	Anti-human IgG or IgM
Suspension array	[47,48]	polymeric	Flow	Covalent and site-specific	1:40	Anti-human IgM or IgG R-phycoerythrin conjugated
Glycan array	[24,49,50,51,52]	monomeric	Static	Covalent and site-specific	1:15	Anti-human IgA, IgG&IgM biotin conjugated; Streptavidin-Alex^555^ conjugated
Glycan array	[53,54]	monomeric	Static	Covalent and site-specific	1:20	Cy3 conjugated anti-human IgG or IgM
Glycan array	[19,41,55,56]	monomeric	Static	Covalent and site-specific	1:20 (up to 1:40)	Anti-human IgA,IgG&IgM or separately all of them biotin conjugated; Streptavidin-europium conjugated
Glycan array	[57]	monomeric	Static	Covalent and site-specific	1:100	Cy3 conjugated anti-human IgG
Glycopeptide array	[58,59,60,61,62]	polymeric	Static	Covalent and (semi-) site specific	1:50	Cy3 conjugated anti-human IgG, IgM, IgA together and separately
Glycopeptide array	[23,25,63]	polymeric	Static	Covalent and site-specific	1:25 (up to 1:3000)	Cy3 conjugated anti-human IgG, IgA and IgM separately (in combination and study dependent)

More recently, new high-throughput platforms have been introduced which are referred to as glycopeptide arrays (Table 1). This array format is characterized by the addition of a carrier protein or polypeptide forming glycoconjugate-based epitopes. Profiling anti-glycan antibodies to glycopeptides on array platforms has been reported for instance by applying bovine serum albumin as carrier protein to epoxide-derivatized slides [61]. In this study neoglycoconjugates were fabricated and carbohydrates synthesized to investigate the antigenicity to anti-glycan antibodies. Another platform utilizes an identified cancer-specific immunodominant glycopeptide epitope in MUC1 [64], a heavily glycosylated mucin known to be associated to several cancer types including breast and ovarian cancer. A synthesized MUC1 peptide was also used as a carrier for the chemoenzymatic synthesis of glycoconjugates (*O*-glycopeptides), on *NHS*-activated glass slides via amine group guaranteeing covalent and site-specific attachment [23,25,63]. Glycan and glycopeptide arrays are optimal glycan-based immunoassays to profile anti-glycan antibodies in high-throughput but concerns still remain because assay dynamics are static, background binding is controversial, and detection of bound anti-glycan antibodies can only be visualized by the use of chemical labels and multiple-step procedures. In parallel to glycan-based arrays, microarray technologies using immobilised lectins for glycomic analysis emerged in the past decade (for review see [65]), but they are beyond the scope of this review.

New technologies in the field of glycan-based immunoassays were introduced which may overcome the previously mentioned limitations. These are glycan-based suspension arrays as well as surface plasmon resonance (SPR) platforms (Table 1). Both technologies are characterized by flow assay dynamics narrowing glycan-antibody interactions more closely to an *in vivo* environment. Recent advances in the field of flow-cytometry enabled a new generation of microbead-based immunoassays, allowing for quantitative simultaneous detection of multiple analytes in a single sample with high sensitivity and reproducibility (for review see [66]). We have utilized this technology to create the first glycan-based suspension array for human anti-glycan antibody profiling [67]. The innovation of this approach is the combination of unique chemically synthesized monobiotinylated glycopolymers [68] used for microbead modifications with the advantages of multiplexed flow-cytometric detection. Optically-encoded microbead-based arrays are characterized by increased control over array preparation, easy reconfiguration of arrays and stability of pre-coated microbeads [69]. Since glycan-based arrays are ideal for screening of very broad glycan libraries, glycan-based suspension array seems to be optimally suited for simultaneous detection of up to several dozens of analytes, thus holds a great diagnostic potential for human serum antibodies in a clinical setting.

Biophysical sensing techniques based on evanescent waves such as SPR have matured to become major tools in protein expression analysis and have also gained considerable momentum in the pharmaceutical industry. Glycan array based on SPR technique allows real-time and label-free detection of carbohydrate-protein binding and to expand the picture of monomeric antigenicity (as in glycan-based microarrays) to a polymeric presentation of glycans. SPR-based measurements are currently possible in a mid- to high-throughput format. As an example, de Boer and co-workers have utilized a SPR array platform which contained 144 glycan structures, released from their natural source [46]. Glycans were covalently and site-specifically attached to epoxide modified surface via fluorescence spacer contained amine group. The chip represented the glycan repertoire of the human parasite *Schistosoma mansoni*. Anti-glycan antibodies of IgG and IgM subclass were recognized in infected and non-infected human serum samples, demonstrating an effective set up of SPR-based screening of anti-glycan antibodies. Another SPR-based glycan assay, composed of a limited number of mannose and galactose derivatives, was constructed showing its feasibility mapping glycan-protein interactions with known lectins (PNA, soybean agglutinin and others) [70].

## 2. TAC in Gynecological Cancers

### 2.1. Tn Antigen

The Tn (*N*-acetylgalactosamine; GalNAcα-O-Ser/Thr, Figure 1) antigen refers to a monosaccharide which is usually attached to the amino acids serine or threonine (or tyrosine in a few cases) of a peptide by a glycosidic bond (forming *O*-glycan). Tn is a cryptic precursor of the T antigen (Core 1) and can be unmasked if cells lose their ability to synthesize Core 1 structure. The expression of Tn was first discovered in 1957 on subpopulations of blood cells characterizing a rare hematological disorder, the Tn syndrome [71]. In the classical work of Springer it was shown that Tn as the truncated form of oligosaccharide chains are abundantly expressed on carcinoma cells [28]. These simple TACA can be carried by cancer-associated mucins and occur in around 90% of breast carcinomas, but are masked in benign and healthy tissues [28,72,73,74,75,76]. Assays based on monoclonal antibodies (mAb) and lectins have shown that expression of Tn in breast cancer is associated with high grade ductal carcinomas [3,75,77]. Its expression was found to significantly predict a shortened 5-year disease free survival, a positive lymph node status and increased combined histological stages [75]. Another study found that Tn antigen expression detected by Tn-specific *Vicia villosa* lectin (VVL-B_4_) in ovarian cancer was correlated with increased malignancy, metastatic progress and low patient survival [2]. Increased Tn antigen expression is also correlated with metastatic potential and poor prognosis in cervical cancers [78,79]. 

Nevertheless, the mechanisms linking Tn antigen expression to cancer progression still remain unknown. Tn on MUC1 was shown to be bound by the macrophage galactose-type lectin on macrophages and dendritic cells [80] and Tn presence may enable the tumor to escape immunosurveilance [81]. Beside its aberrant function the genetic basis causing Tn appearance on *O*-glycoslyated proteins is still under investigation. It is becoming evident that the loss of functional COSMC is one molecular explanation for the increased Tn expression on human cancer cells [82]. COSMC is an essential chaperone for correct protein *O*-glycosylation and loss of COSMC is associated with loss of T-synthetase and increase in Tn antigen [83]. In cervical cancer a deletion of functional allele (LOH) leads to complete absence of COSMC and increased expression of Tn and sTn [82]. 

Early pioneering work by Springer and colleagues reported experiments for long-term anti-carcinoma vaccination and treatment of breast cancer [84] without delivering additional proof. Tn expression has been linked to tumor progression and targeted cancer treatment which has been used for anti-cancer vaccination and treatment of breast cancer [64]. Carbohydrates alone do generally not activate T lymphocytes and have therefore reduced immunogenicity [85]. The increased immunogenicity can be achieved by linking Tn to carrier protein such as keyhole limpet haemocyanin (KLH) [86], MUC1 peptide [87,88], or the use of immunological adjuvants such as saponin [89], forming glycoconjugates to generate anti-glycan antibodies. A pilot study in a cohort of epithelial ovarian, fallopian tube, and peritoneal cancer showed an induced prevalently IgM-antibody response to a heptavalent vaccine including Tn and Tn-MUC1. Only Tn-MUC1 revealed both IgM and IgG response [90]. These observations are in concordance with another more recent study where natural anti-glycan antibodies were detected using a glycopeptide array [25]. This clearly indicates recognition of Tn by the cognate immune system. Despite the chemical simplicity of Tn antigen, its antigenetic structure is considered to be rather complex and recent data suggest that Tn antigen antibody binding capacity is determined by the peptide context of Tn antigen [91]. This finding is of importance due to previously used Tn-peptide vaccines and should therefore be considered. In addition, first promising clinical trials in prostate cancer patients showed that only cancer-specific Tn expression on tumor cell surface enables targeting and site-specific treatment [86].

### 2.2. Sialyl-Tn Antigen

STn antigen tissue expression and its presence in blood were found in various gynecological cancers originating from the ovary, cervix, endometrium and vulva. The transfer of sialic acid in α2,6-linkage to Tn structure usually terminates the further elongation of oligosaccharide. Therefore, sialyl-Tn (sTn, Neu5Acα2-6GalNAcα-O-Ser/Thr, Figure 1) expression leads to a shortening of *O*-glycan chains [2]. STn displays restricted expression in normal tissues [72,92], but can be detected at various frequencies in almost all kinds of carcinomas, even more frequently in adenocarcinomas. At least 25-30% of breast cancers are sTn-positive [93] and overexpression of sTn occurs in almost 40% of breast cancers [94]. STn expression was found to be higher in ovarian cancer patients which were associated with shorter survival [95]. There is increasing evidence that sTn expression is similarly associated with survival in breast cancer [96,97,98,99], potentially as a short-term outcome [97]. In node-positive breast cancer patients sTn expression was also correlated with a lack of response to adjuvant chemotherapy [98]. Using immunohistochemical staining and anti-sTn monoclonal antibody (TKH-2), the disaccharide was detected in a majority of ovarian and cervical cancers with no positive match in remaining cancer types, benign, and normal controls [79]. A reduced number of tissue sTn positive samples also showed detectable levels of serum sTn. Using the same mAb directed to sTn, another study found detectable levels of sTn in serum of ovarian cancer patients which significantly correlated with increased malignancy, metastatic progression and low patient survival [100]. An increased sTn expression in ovarian carcinoma cells was detected when primary tumors were compared with metastatic lesions [101]. Another study of the same investigators, conducted on tissue samples from 45 patients, confirmed that sTn is widely expressed in ovarian carcinomas and related metastases, but could not verify sTn expression to be predictable of disease outcome [102]. There is a clear indication that sTn expression in tissue and blood serum correlates with tumor progression in breast and ovarian cancer. The mechanisms, underlying the appearance of this *O*-glycan in several types of carcinomas is still unknown, as is its varied tissue expression. One possible explanation suggests an increased gene expression of *ST6GalNAc-I* glycosyltransferase, the enzyme which transfers sialic acid to Tn antigen, thus creating Neu5Acα2-6GalNAc, which is the sTn epitope [103]. It has been demonstrated that this is not the case in colon cancer because ST6GalNAc-I activity was not elevated in cancerous colonic tissues as compared to normal mucosa. In contrast, sTn was detected in cancer cells and was absent in normal controls [104]. The transfection of *ST6GalNAc-I* and reconstitution of sTn expression was performed in breast cancer cells and demonstrated that the expression of RNA-encoding *ST6GalNAc-I* and the expression of sTn are directly linked [93]. The discrepancy to observations in colon cancer were explained by reduced sialic acid *O*-acetylation, unmasking sTn for mAb recognition [105]. STn antigen is usually present on *O*-glycosylated proteins such as MUC1 [106], CD44 [107], and MUC16 [108]. It has been suggested, that altered glycosylation of these molecules may influence adhesion and migration (motility) of cancer cells. Namely, sTn expression in breast cancer cells is sufficient to modify biological features, decreasing adhesion and increasing migration and tumor growth [109,110]. CD44 as the main hyaloronan (nonsulfated glycosaminoglycan) receptor appears to play an important role in mediating the binding of tumors to the extra-cellular matrix (ECM) [111,112]. STn as a classical TACA has also been demonstrated to be widely recognized by naturally occurring antibodies not only in cancer patients, but in healthy controls. In a study on 106 healthy donors which investigated the binding to anti-glycan antibodies on a glycan array, high-levels of anti-sTn antibodies were found [52]. In a study of ours, using the same glycan array, we also observed detectable levels of anti-glycan antibodies to sTn in healthy and non-mucinous ovarian cancer patients without significantly distinguishing these two groups [24]. In addition, our custom-made suspension array [48,67] detected anti-sTn antibodies that significantly correlated with clinico-pathological characteristics of gynecologically investigated samples (data not published). Glycopeptide array incorporating sTn-MUC1_60mer_ glycopeptides revealed high levels of anti-sTn antibodies significantly associated with reduced incidence and increased time to metastasis in breast cancer patients [23]. In so far as elevated levels of sTn in breast cancer are associated with poor prognosis, these findings on anti-sTn antibodies suggest their evident role in anti-cancer immune response. Nevertheless, a direct proof showing correlation of anti-sTn antibody levels in patient sera and sTn expression in matched tissue samples is still needed. 

### 2.3. T Antigen

Another *O*-linked disaccharide with a potential tumor association is T antigen (Galβ1-3GalNAcα-O-Ser/Thr, T, Figure 1) also referred to as Thomsen-Friedenreich antigen (TF) or Core 1 glycan. T antigen, initially described on glycophorins on red blood cells, is the cryptic precursor of Core 2 *O*-glycans, which can be unmasked if cancer cells lose their ability to synthesize Core 2. Namely, T antigen is unsialylated Core 1.

It is known that TF occurs in ~90% of all human cancer cells including precancerous conditions [113]. Springer and co-workers first showed immunohistochemically (using peanut lectin (PNA) and natural human anti-T antibodies), that breast cancers, but not benign tumors or normal mammary cells, expressed T antigen [114,115]. Their further investigations, conducted on primary invasive breast ductal cancer tissues using mAbs and lectins, demonstrated, that T epitope expression showed no statistically significant association with prognostic factors [75,116]. Also, no prognostic value of T antigen in breast carcinomas was detected by PNA staining [117]. With the use of T-specific mAbs, however, a worse prognostic impact of T-expression in breast cancers was found [118]. In contrast, another study [119] using mAbs showed the correlation of T antigen expression in breast cancer with a better prognosis, which was opposite to gastrointestinal, lung or cervical cancers. A recent study demonstrated immunohistochemically that T antigen was significantly expressed in normal epithelium compared to CIN I, CIN II and invasive cervical cancer [120]. These contradictory results may be related to technical and experimental limitations and use of different probes [121,122]. Also, small haptens (as disaccharide T) are differently recognized by antibodies in natural microenvironment (being numerously attached to protein backbone) and in immunoassays (ELISA, PGA) [49,123]. In ovarian cancer T antigen specific expression was described depending on the ovarian cancer histotype [95,124,125].

There are several indications that TF is expressed in cancerous cells and therefore refers to TACAs. Nevertheless, only recent studies have started to investigate its molecular function. There are some indications that TF antigen may play a role in oncogenic proliferation [86,116]. No certain mechanism of TF antigen action has been described, on the other hand, although TF could be a good ligand to galectin 3 [126,127], which has been reported to promote breast cancer metastasis by adhesion to endothelial cells. TF binding abilities of galectin 1 and 3 were recently investigated using crystallization studies and SPR assay demonstrated increased affinity of galectin 3 over galectin 1, identifying a unique motif for TF binding [128]. The results additionally indicate that TF could be recognized by galectins, a family of beta-galactoside binding proteins involved in modulation of cell-cell and cell-matrix interactions.

The cancer-specific association of TF shows that this glycan would probably have clinical applications. For instance, application of anti-TF mAb successfully inhibited lung metastasis in mice and improved prognosis in a mouse breast cancer model [129]. In a vaccination study using TF conjugated to KLH in combination with an adjuvant therapy in ovarian cancer, a clear immune recognition of TF-glycoconjugates were found with anti-glycan antibody responses of IgM (n=9), IgG and also IgA subclasses [130]. The immune response was also observed in a more recent study in prostate cancer patients [86]. 

### 2.4. Lewis Structures

Lewis structures are blood group antigens which contain fucose. This monosaccharide is either α1-3 or α1-4 linked to *N*-acetylglucosamine (GlcNAc). The histo-blood group Lewis antigens are found in most human epithelial tissues, as far as they are terminal parts of glycolipids and glycoproteins. There are two types of Lewis glycans: (A) Le^a^, sLe^a ^and Le^b^, all derived from type 1 structures (Galβ1-3GlcNAc, Le^c^) and their positional isomers (B) Le^x^, sLe^x ^and Le^y^, derived from type 2 chains (Galβ1-4GlcNAc) with substitutions by fucose and sialic acid (Figure 1). 

In healthy individuals, Le^a^ and Le^b^ (type 1) as well as Le^x^, Le^y^, sialyl-Le^x^, sulfo-Le^x ^(type 2)- Lewis antigens are normally expressed as terminations of numerous glycoconjugates and are characterized by an overall lack of autoantibodies [52]. However, Lewis structures have been widely reported to be associated with cancerous conditions. In general, reduced expression of type 1 Lewis antigens and increased expression of type 2 antigens have been observed more or less consistently in carcinogenesis [131,132]. Loss of Le^b^ and, to some extent Le^a^, in invasive ductal carcinomas of the breast compared to normal and benign tissues was correlated with the grade of malignancy [133,134]. Expression of sLe^x^ (type 2 structure) on the other hand, was increased in patients with advanced and recurrent breast cancer [135,136].

Breast cancer and 80% of ovarian cancers are of epithelial origin and the most characteristic TACAs in these cancers are sialylLe^x^ (sLe^x^), sialylLe^a^ (sLe^a ^), and Le^y^, occurring in most human epithelial tissues [99,131,132,137,138]. In cancer, overexpression of sLe^x^ or sLe^a^ antigens and the general increase in sialylation are typical alterations [139,140]. When found at the surface of carcinoma cells, they are usually associated with a poor prognosis in tumors of certain type, stage and grade, and reduced overall survival [98,139]. The expression of sLe^x^ in breast cancers is also considered to be an independent prognostic indicator of survival regardless of the primary tumor and lymph node involvement [141]. Elevated sLe^x^ expression was found in patients with metastases compared to those without [136], and sLe^x^ was increased in serum of patients with advanced breast cancer [135,136]. Combined detection of CA15-3, the most commonly used breast cancer tumor marker, and sLe^x^ in serum improved the effectiveness of monitoring metastatic breast cancers (78.5% *versus* 61.5%, when measured by CA15-3 alone) [142]. Using high-performance liquid chromatography (HPLC) and mass-spectrometry (MS) a number of sLe^x^ bearing proteins were identified as predictors of breast cancer progression in patients with advanced breast cancer [135]. The *N*-glycan profiles from healthy and advanced breast cancer groups were significantly altered, as highlighted by an average 2-fold increase of sLe^x ^in the serum of cancer patients. This preliminary finding indicates that elevated levels of this potential glycan marker might be a better marker than CA 15-3. Controversially, an immunohistochemical evaluation of a cohort of 127 patients with primary breast cancer of various stages and grades has demonstrated, that sLe^x ^expression was not correlated with prognosis and survival [143]. Studies conducted on primary ovarian carcinomas and metastatic lesions, demonstrated that Le^y^ and sLe^x^ are widely expressed in both, but their expression did not seem to correlate with long- or short-term survival [101,102]. The characteristic changes in total serum *N*- glycans from patients with advanced ovarian cancer of different type, were examined by HPLC, weak anion exchange HPLC and MS [144]. These changes included increases in levels of core fucosylated, agalactosyl biantennary glycans, presented on IgG heavy chains, and in levels of sLe^x^, linked to acute-phase proteins, such as haptoglobin, α1-acid glycoprotein, and α1-antichymotrypsin.

The mechanisms defining sLe^x^ and sLe^a^ malignancy are more explained than in other TAC. SLe^x ^and sLe^a^ are known ligands for E-selectin [99,100] and are known to facilitate cancer cell metastasis, mediating their extravasations from blood to peripheral tissues via E-selectin, expressed on vascular endothelium [145,146,147,148,149,150]. Some of these investigations were conducted on the breast cancer model and were supported by clinicopathological data [131,146]. Thus, both sLe^x^ and soluble E-selectin were significantly elevated in the serum of breast cancer patients with advanced and recurrent disease [151]. Both P- and E-selectin expression was significantly elevated on endothelial cells of breast cancer patients [152]. The recent study confirmed E-selectin- driven mechanism of sLe^x^ action in carcinogenesis [153]. Importantly, the authors demonstrated that glycosylation profiles differ between estrogen receptor (ER)-positive and ER-negative breast cancers with higher incidence of sLe^x ^in ER-negative breast tumors due to significantly elevated expression of corresponding glyco-genes. SLe^x^ expression had no influence on the survival of patients regardless of their ER-positive or ER-negative status. However, high expression of sLe^x^ in ER-positive tumors correlated with bone metastasis – the expression site of E-selectin, the receptor for sLe^x^. The authors suggest that selectins may promote metastasis in breast cancer through protein-associated sLe^x^ and heparansulfate (HS) glycosaminoglycans, as their expression was similarly increased in ER-negative tumors and they may engage with selectins (L/P-selectin) via various microdomains [154]. Notably, selectin affinity depends strongly on sLe^x^ microenvironment: for example, E-selectin weakly binds to sLe^x^-containing glycolipids [155], but has a strong affinity for *O*-glycan associated sLe^x^, expressed by neutrophils [156]. 

Le^y^ (difucosylated Lewis antigen type 2) is overexpressed in 60 to 90% of cancers of epithelial origin, including breast and ovarian cancer [157] and correlated in lymph node-negative breast cancers with poor prognosis and decreased patient survival [158]. Le^y^, being overexpressed in 75% of ovarian carcinomas, correlated with highly malignant phenotype [159]. These findings indicate the impact of fucosylation in carcinogenesis. Le^y^/Le^b^ – induced drug resistance in ovarian cancer cell lines has been reported [160]. Overexpression of Le^y^ presumably confers cell adhesion-mediated drug-resistance to apoptosis in ovarian cancer cells by up-regulation of TOPO I and TOPO II β proteins [161]. Le^y^ structures were found to be associated with CD44, and it was shown that overexpressed Le^y ^strengthens CD44 mediated adhesion and spreading of ovarian cancer cells [162].

### 2.5. Glycoshingolipids

#### 2.5.1. Gangliosides

Glycosphingolipids are glycolipids containing the amino alcohol sphingosine. Gangliosides, as one out of three subgroups of glycosphingolipids, are ubiquitous membrane-associated glycolipids containing one or more neuraminic acids. Gangliosides are derived from lactosylceramide and additional glycan residues – Neu5Ac, GalNAc and Gal (Figure 1). There are a-, b- and c-series of gangliosides synthesized by sequential activities of sialyltransferases and glycosyltransferases. In adults, they are normally thought to be restricted to the central nervous system [163] but gangliosides seem to also play important roles in breast cancer progression and metastasis [164,165]. A prominent candidate is the disialoganglioside GD2 (Figure 1). A very recent study identified ganglioside GD2 as potential breast cancer stem cell marker and demonstrated its involvement in carcinogenesis. These cells bearing GD2 were capable of forming mammospheres and initiating tumors. In addition, gene expression analysis revealed that GD3 synthase gene expression is responsible for the presence of GD2 [166]. Gangliosides GM3, GD3 (Figure 1), as well as unusual *O*-acetylated gangliosides (9-*O*-acetyl-GD3, 9-*O*-acetyl-GT3), were found to be overexpressed in about 50% of breast cancer patients [167]. Another ganglioside, *N*-glycolyl-GM3, not found in normal human tissues, was detected in primary tumors of FIGO Stage II breast cancer [167,168]. Overexpression of monosialoganglioside GM3 and disialoganglioside GD3 (Figure 1) may correlate with higher malignancy of breast cancer cells by enhancing cell proliferation and migration [165]. Total levels of gangliosides were also observed to be increased in serum and ascites of patients with advanced ovarian cancer [169], indicating the process of shedding or release of gangliosides, which is associated with cancer progression. It was suggested, that shedding of gangliosides into the local tumor microenvironment contributes to tumor strategies to evade immune recognition, thus high concentration of circulating gangliosides is associated with poor prognosis. The mechanism of evasion of the innate immune response may be based on inhibition of antitumor natural killer T (NKT) cell response. Such action of circulating ganglioside GD3, purified from ovarian cancer-associated ascites was recently shown by Webb and co-workers [170].

#### 2.5.2. Globosides

Globo H and stage-specific embryonic antigen 4 (SSEA 4) have the same precursor, stage-specific embryonic antigen 3 (SSEA3), and therefore sharing a structural motif (Figure 1). Both glycosphingolipids, Globo H and SSEA4, are only different by their terminating monosaccharide, α1-2-linked Fucose and α2-3-linked Neu5Ac, respectively (Figure 1). These glycans are of importance because both have been described in association with cancer.

Globo H, a fucosylgalactosylgloboside, was originally characterized by Hakomori and co-workers in a human breast cancer cell line [171]. Expression of Globo H (both as GSL and glycoprotein) in 143 cases of breast cancer was not correlated with patient survival [172]. Using glycan array a significant difference in anti- Globo H antibody levels in serum of breast cancer patients and healthy donors was demonstrated, and the increase of Globo H in breast cancer stem cells was associated with the disease progression [53]. Globo H was also found to be expressed in ovarian cancer [2,173]. 

## 3. Conclusions and Discussion

Despite immense progress in research of cancer-associated glycans during the past three decades, this area of glycobiology and molecular oncology still opens broad possibilities for scientific discoveries. A number of regularities of cancer-associated glycosylation were clearly defined. We have mentioned and discussed the main known cancer-associated glycans involved in breast and ovarian cancer. For example, breast cancer cells express truncated Core 1-based glycans including T, Tn and sTn antigens instead of elongated *O*-chains. The expression of sialyl-Lewis antigens is also altered and complex gangliosides are overexpressed in invasive ductal carcinomas. In ovarian cancer, sLe^x^/sLe^a ^and their analogues as well as sTn and Le^y^ have been correlated with metastatic potential and patient survival. However, lectins and most monoclonal antibodies, used in classical studies, are not strictly specific for certain carbohydrate structures but expose some degree of cross-reactivity (for review see [121]). Thus, novel glycan cancer associated epitopes, especially combined glyco-peptide and -lipid epitopes, presumably specific for certain cancer types/stages/grades are still in the process of identification. We need to know more about TACA, the structure of TACA-bearing glycoconjugates, the role of their molecular architecture *in vivo* for immune recognition, alterations of glycosyltransferase expression in cancer, and, finally, the molecular mechanisms of their action. Currently, the mechanisms defining malignancy are fairly understood only for sLe^x^ and sLe^a^, as these TACA mediate binding of tumor cells to microvascular endothelium through E-selectin expressed on the endothelial cells. The biochemical mechanisms of action of other cancer-associated glycans in cancer progression are still under evaluation [5,9,11].

Naturally occurring anti-glycan antibodies have been demonstrated to recognize TACA in many different types of malignant diseases as observed through glycan-antibody interaction. In the past decade, high-throughput glycan arrays as well as glycopeptide arrays have been increasingly used. These approaches already contribute to biomarker research in breast and ovarian cancer, and will be powerful tools in the future, when increasing efficiency, sensitivity and preciseness will allow cancer diagnostics and therapeutics of high-sensitivity.

Despite detected TACA-specific interactions with antibodies, it is crucial to conclude that TACA are specifically expressed or shed by cancer cells and the direct proof of their presence is often missing. It is therefore required to test matched serum samples from the same patients using alternative methods. Matched tissue samples of normal and cancer patients can allow the identification of TACA directly. This can easily be achieved by standard immunohistochemistry using mAbs or lectins. The latter are known to bind various glycan structures sharing carbohydrate motifs or epitopes (for review see [174]). 

Other possibilities are the identification of glycan structures by MS-based profiling and the analysis of glycan complements in plasma and tissues, which allows for the comprehensive analysis of membrane protein glycosylation*.* The high-throughput glycan profiling by MS requires only minute volumes of patient serum, thus representing an essentially non-invasive diagnostic method. This highly sensitive method in contrast to glycan-based immunoassays, detecting anti-TACA antibodies (glycan and glycoconjugate based platforms), can be used for direct glycomic mapping and as a proof of glycoarray-based findings.

In breast cancer research a sensitive specific MS (MALDI-TOF MS)-based glycomic profile was performed to analyze *N*-glycans in serum of control as well as early- and late-stage breast cancer patients. Various MS-based technologies in combination with other methods (high performance liquid chromatography, capillary electrophoresis) were also consistently used for the investigation of gynecological cancer associated glycan alterations over the past decade [93,135,144]. Differences in glycomic profiles revealed a substantial increase of fucosylation (both in core structures and the branched segments) in cancer patients, whereas various sialylated structures in serum presented a less clear picture. In one study changes in relative intensities of eight glycans are characteristic of breast cancer, whereas some other glycan structures might contribute additionally to distinctions in the recognizable patterns [175]. 

For the establishment of potential new tumor markers and understanding the functional role of anti-glycan antibodies, validation of glycan-based techniques still remains complex and complicated due to the limited number of samples, the heterogeneous pathology of cancer diseases - especially in epithelial ovarian cancer, the absence of independent glycan-based immunoassays, and the fabrication of glycans itself. We have profiled our previously detected top candidate P_1_ trisaccharide [24] in an independent cohort using an independent glycan-based immunoassay [48]. We have also utilized ABO blood group antigens, A and B trisaccharides, to study the reproducibility of different glycan-based immunoassays, presentation of glycans, the use of various types of glycoconjugates, and assay dynamics. We found a high correlation of anti-glycan antibody levels in ABO blood group antigens using three methods: ELISA, printed glycan and suspension array. Interestingly, in terms of P_1_ trisaccharide correlation between methods decreased from moderate to low indicating that presentation of glycan, antigen/ antibody ratio, assay conditions and detection technique is crucial. This further indicates that the glycan-antibody interaction of interest has to guide the assay selection [48]. 

In conclusion, as can be observed from the literature and from our own research, TAC could be excellent biomarkers for cancer. Also, the immune response in cancers is clearly and predominantly related to the glycan, or combined glycopeptide/glycolipid epitopes – whether expressed as glycoproteins or glycolipids. Recent advances in glycomics enabled development of novel high-throughput experimental and technical platforms for TAC research, which was classically based on immunohistochemical studies. These analyses of simple TACA unraveled the main features of aberrant cancer-associated glycosylation, but could not reveal the entire information concerning specific and complex modifications in total glycoconjugates during carcinogenesis. Nowadays there are various platforms available profiling human anti-glycan antibodies to identify potential glycan-antibody interactions. These novel approaches include printed glycan array, glycopeptide arrays, bead-based suspension array, and SPR array. These platforms show great potential as usable and powerful tools for specific glycan biomarker discovery as they offer the potential to profile hundreds of array elements simultaneously, require minute amounts of reagents and are suitable for large scale sample analysis. Finally, these developing detection methodologies may be of great value in clinics for diagnostic/ prognostic purposes and for enabling patient-tailored treatments.
